# Writing directly to patients, the time is now—but how?

**DOI:** 10.1093/rheumatology/keae093

**Published:** 2024-02-09

**Authors:** Charlotte A Sharp, Karen Staniland, Brian McMillan, Jill Firth, William J Gregory, Elizabeth M MacPhie, William G Dixon

**Affiliations:** Division of Musculoskeletal and Dermatological Sciences, University of Manchester, Manchester, UK; Kellgren Centre for Rheumatology, Manchester Royal Infirmary, Manchester University NHS Foundation Trust, Manchester, UK; Division of Musculoskeletal and Dermatological Sciences, University of Manchester, Manchester, UK; Centre for Primary Care and Health Services Research, University of Manchester, Manchester, UK; Pennine MSK Partnership, Integrated Care Centre, Oldham, UK; Rheumatology, Northern Care Alliance NHS Foundation Trust, Salford, UK; Faculty of Health and Education, Manchester Metropolitan University, Manchester, UK; Rheumatology, Lancashire and South Cumbria, NHS Foundation Trust, Preston, UK; Division of Musculoskeletal and Dermatological Sciences, University of Manchester, Manchester, UK; Rheumatology, Northern Care Alliance NHS Foundation Trust, Salford, UK

How would you feel if your hospital specialist and primary care clinician discussed your health in front of you without including you in that conversation, using words you couldn’t understand? Historically, secondary care healthcare professionals (HCPs) have written a summary of the outpatient clinic visit to primary care HCPs, describing patients in the third person. In the early 2000s, the National Health Service (NHS) stated that it was the patient’s right to be copied into outpatient clinical letters regarding their healthcare [1], which is now standard practice. Letters copied to patients are often written in medical language; typically containing technical detail, jargon and Latin abbreviations, which may be difficult for patients to understand [2]. For example, ‘chronic’ is often interpreted by lay people as being particularly severe rather than ‘long-lasting’, which is the traditional medical interpretation of the word [3].

The aim of writing directly to patients is to inform, engage and remind patients of the agreed upon care plan. Potential additional benefits of this more inclusive communication approach include improved patient understanding and experience, supported shared decision making, more patient-centred care, better patient–clinician relationships, the potential to reduce complaints due to improved patient understanding and a reduced burden on general practitioners to ‘translate’ clinic letters for patients [3]. The EULAR and ACR have endorsed a common-language description of the term ‘rheumatic and musculoskeletal diseases (RMDs)’ [4]; we are not aware of international agreements on how summaries of rheumatology clinic consultations should be communicated with patients. Last year in the UK, existing guidance from 2018 [5] was endorsed by NHS England, advising HCPs to write clinic letters directly to patients and copied to primary care [6].

Practice varies internationally and there is very little evidence regarding rates of uptake, reasons behind these rates and patient preferences for terminology. In some places patients receive no communication, some have access to the clinic notes via patient portals such as EPIC’s MyChart, some write to primary care and send a copy to patients and others write the letter directly to patients, copying in other relevant HCPs. In the UK, we are now 5 years on since the 2018 guidance, and current evidence [7], and our (anecdotal) understanding, is that only a minority of HCPs have adopted the practice. Uptake appears to vary between and within rheumatology departments across the UK nations, and indeed between the authors of this editorial. Some clinicians write directly to patients to report investigation results, while retaining the more traditional approach of writing clinic letters to HCPs. Specific to rheumatology, there are no easily identifiable published reports about how well rheumatology terminology is understood by patients, although evidence is available for other specialties [8]. There is little evidence available regarding the feasibility and acceptability of writing to patients seen in a rheumatology clinic.

While writing directly to patients may be viewed as a common-sense approach to improving patient understanding and the clinician–patient relationship, why is the uptake so varied? Using medical language is an important part of our clinical decision-making techniques, taught early on in our training, with the aim of helping us to digest and assimilate complex information into memorable storylines or illness scripts [9]. Having used the medical language to help with our clinical reasoning, we then need to make this information accessible to our patients and to other HCPs involved in their care.

HCPs may worry that clear communication between clinicians may be lost without the aid of precise medical terminology and that translating this into plainer language may take a lot of time. We do not advocate writing multiple letters for different audiences for the same encounter and argue that it is possible to meet the needs of all stakeholders without jeopardizing clinical accuracy or making unmanageable demands on HCPs’ time. For example, a list of medical diagnoses may be provided at the top of the letter, with the main text containing a summary of the consultation that is understandable by the patient. Messages and requested actions for primary care colleagues need to be communicated clearly and included in the same correspondence. Messages to help motivate and engage patients, and remind them of the agreed upon care plan, should also be included. Such an approach may save administrative time spent correcting artificial intelligence / human-generated typographical errors and save time spent in future contacts with patients, who should be better informed about their long-term condition.

From a cultural perspective, changing the language used might be seen as representing a shifting of power, threatening traditional medical hierarchies and paternalistic approaches to healthcare. In addition to the use of medical language to communicate scientific observations between HCPs, showcasing knowledge by using this language may be seen to bring with it a degree of cultural capital. Perhaps more pressing than these (arguably antiquated) perspectives is the challenge of altering often ingrained and ‘second nature’ practices of using medical terminology when writing directly to other HCPs. Learning to rephrase and simplify language may take time, be easier for those with more refined linguistic skills or be particularly challenging for those practicing medicine in a second language.

Implementing a change in practice to writing directly to patients involves challenges other than the linguistic skills of the HCP. We need to encourage patients to read and engage with the contents of clinic letters; a difficulty faced by a member of this team when implementing this practice in a deprived area with wide variations in health literacy. Low health literacy is linked to poorer health outcomes, and there is a current mismatch between materials designed for patients and their health literacy. Writing directly to patients requires acknowledging this variation in health literacy. Correspondence written between HCPs, and which consequently side-lines the patient or ignores patients’ individual communication needs, does not address this variation.

Despite these challenges, and the important context of stretched services and HCPs working within those services, writing clinic letters directly to patients is a step that we all need to take. What is needed to support rheumatology HCPs to take the leap from the comfort of traditional communication talking about patients to writing directly to them? When discussing this within our region, many colleagues wanted to write directly to patients and voiced their hesitancy in doing so because of a lack of confidence in making this switch. They expressed a desire to be provided with support and example letters to help them make, what may seem, a daunting transition. We are working to understand the issues involved in writing directly to patients and to develop resources to support the multidisciplinary team to write clinic letters directly to patients [10]. We would advocate that our secondary care colleagues prepare for this challenge, encouraging them to discuss the practice with patients and seek engagement and patient experience feedback to inform improved written communication [11].

Clinic letters involve multiple stakeholders. We argue that this should include the patient at the centre, fulfilling their information needs without compromising the communication of key messages and actions required of all stakeholders. Writing in medical language will, of course, feel more natural to HCPs than translating this into plain language. But if we as clinicians find it hard to put medical terminology in simple language, how can we possibly expect patients to understand what we write? The question is not *whether* we should write directly to patients, but *how*.

## Data Availability

No new data were generated or analysed in support of this research.
